# Allergic shiners in a patient with cough-variant asthma: a case report

**DOI:** 10.1186/s13256-022-03423-6

**Published:** 2022-05-28

**Authors:** Mahsa Rekabi, Nasim Raad, Atefeh Abedini, Sepideh Darougar, Ali Akbar Velayati

**Affiliations:** 1 Pediatric Respiratory Diseases Research Center, National Research Institute of Tuberculosis and Lung Diseases (NRITLD), Shahid Beheshti University of Medical Sciences, Tehran, Iran; 2 Chronic Respiratory Diseases Research Center, National Research Institute of Tuberculosis and Lung Diseases (NRITLD), Shahid Beheshti University of Medical Sciences, Tehran, Iran; 3 Department of Pediatrics, Tehran Medical Sciences Branch, Islamic Azad University, Tehran, Iran

**Keywords:** Cough-variant asthma, Chronic cough, Asthma, Allergic shiners

## Abstract

**Background:**

Chronic cough, with a duration of coughing of more than 8 weeks in adults, affects 5–10% of the general population. One of the most common causes of chronic cough is cough-variant asthma, which accounts for approximately one-third of cases. This phenotype of asthma is characterized by extreme sensitivity of the neuronal pathways mediating cough to environmental irritants, which results in an urge to cough. This case is an example of cough-variant asthma presenting with allergic shiners due to her severe cough.

**Case presentation:**

A 38-year-old Iranian woman, who was well before the start of the coronavirus disease 2019 pandemic, presented with a nonproductive hacking cough that had begun after excessive use of antiseptic solutions. The only positive finding on physical examination was a reddish-purple rash on and around the eyelids mimicking a heliotrope rash, which had probably evolved due to the severity of the cough. The results of the pulmonary function test were within normal limits. Methacholine challenge test and chest x-ray were both normal. Chest high-resolution computed tomography revealed hyperinflation and tree-in-bud opacities. All other laboratory tests were normal. Because of the reversibility in her pulmonary function test, despite normal baseline parameters, asthma treatment was initiated, resulting in disappearance of the cough and the eye discoloration, being indicative of the correct diagnosis and proper treatment.

**Conclusion:**

Patients with cough-variant asthma may often have no other classic symptoms of asthma other than cough.

## Introduction

Chronic cough by definition is a disabling complaint affecting 5–10% of the general population [1]. It is considered chronic when the duration is longer than 8 weeks in adults and adolescents older than 14 years old [2], and interferes with normal activity and daily life [3]. One of the most common causes of chronic cough is cough-variant asthma, a specific and atypical asthma phenotype, which accounts for approximately one-third of cases [4]. This phenotype of asthma is characterized by airway hyperresponsiveness, eosinophilic inflammation, and bronchodilator responsiveness without clinical manifestations such as wheezing and dyspnea [5]. Patients often show extreme sensitivity of the neuronal pathways mediating cough to environmental irritants such as perfumes, air pollutants, bleaches, and cold air, which results in throat irritation and the urge to cough [1]. Diagnostic tests such as spirometry (before and after bronchodilator) and methacholine challenge tests help to assess bronchial hyperreactivity and lung mechanics, although these methods do have imperfections and should be interpreted only in the context of the patient’s clinical presentation [6]. Early introduction of inhaled corticosteroids may prevent progression of cough-variant asthma to classic asthma [7]. In intractable cases, treatment of the existing gastroesophageal reflux may prove to be helpful [7].

This case is an example of cough-variant asthma with atypical clinical manifestation, presenting with chronic severe dry cough leading to red–purple discoloration of both eyelids, which disappeared after an optimal asthma management plan was put in place.

## Case report

**Fig. 1:**
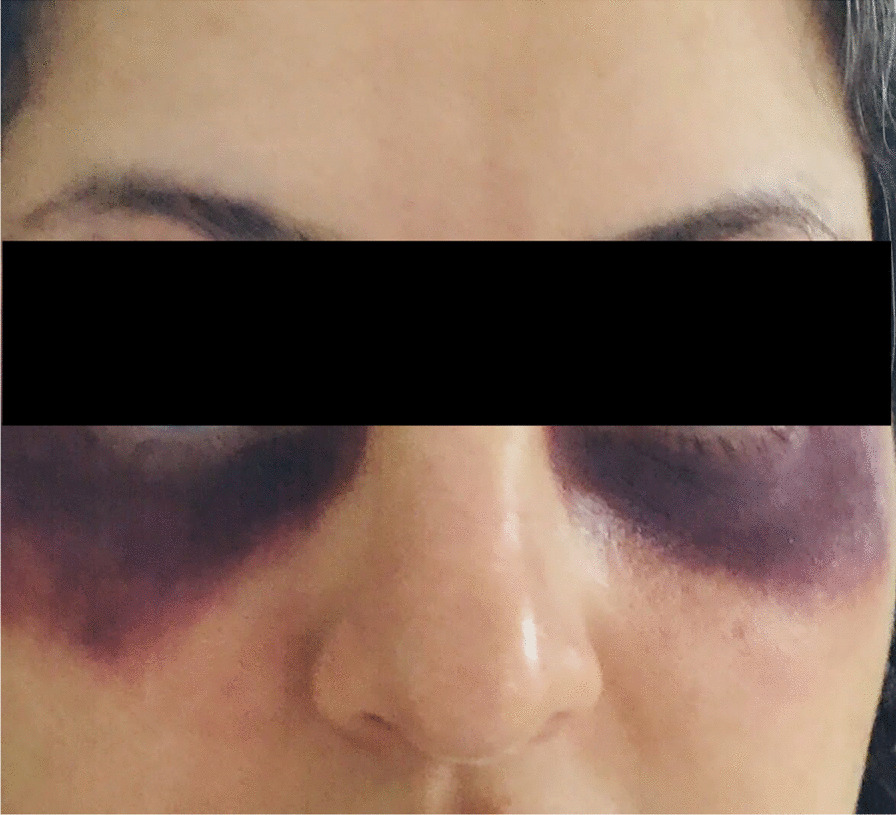
The patient with severe cough and red–purple discoloration of eye contours before treatment

A 38-year-old Iranian woman, who was well before the initiation of the COVID-19 pandemic, presented with a nonproductive hacking cough that had begun after excessive use of antiseptic solutions in order to prevent coronavirus infection. The intermittent severe cough had lasted several days and was aggravated by exposure to perfumes, strong odors, and spices. The symptoms had persisted for approximately 3 months before the patient sought professional medical assistance. She had used different antitussive medications without an optimal response. Her medical history was unremarkable except for allergic rhinitis. There was no history of gastroesophageal reflux disease, chronic sinusitis, tuberculosis, any use of medications, or cardiopulmonary diseases. She was a nonsmoker and a housewife. She did not have any toxic occupational exposure; however, she had overused antiseptic solutions to prevent coronavirus infection for the past 2 years. Physical examination was normal with no extraordinary audible sounds including wheezing or rhonchi. The only positive finding on physical examination was a reddish-purple rash on and around the eyelids mimicking a heliotrope rash, which had probably evolved due to the severity of the cough (Fig. 1).

The results of the pulmonary function test were within normal limits at the first step, but after using a bronchodilator, a substantial increase from baseline was noted in FEV1 (Table 1).

**Table 1:** Patient’s pulmonary function test (pre and post bronchodilator)

Parameter	Pre	Pred	% Pred	Post	% Pred
Best FVC	2.82	3.27	86	2.86	101
Best FEV1	2.34	2.82	83	2.36	101
FEV1/FVC%	82	81	101	81	99
PEF	6.45	6.63	97	7.33	114
FEF 25–75%	2.61	3.62	72	2.38	91

FVC: Forced Vital Capacity; FEV1: Forced Expiratory Volume in the first second; PEF: Peak Expiratory Flow; FEF: Forced Expiratory Flow

Methacholine challenge test and chest x-ray were both normal. Chest HRCT revealed hyperinflation and tree-in-bud opacities. The patient was admitted and underwent complete diagnostic work-up. Sputum was evaluated for bacteriology and sensitivity, with negative results for specific pathogens including *Mycobacterium tuberculosis*. CBC revealed mild hypochromic microcytic anemia. cANCA and pANCA were both negative, and vitamin D3 was mildly insufficient (25 ng/ml). PT, PTT, and INR were within normal limits (Table 2).

**Table 2:** Patient’s laboratory data

Test	Results
WBC	11.33 × 10^3^ cells/μl
RBC	3.94 × 10^6^
Hg	11 g/dl
Hct	34.2%
Plt	263 × 10^3^/μl
Neut	84.6%
Lymph	11%
Mono	4.3%
cANCA	1.4 μ/ml
pANCA	1.3 μ/ml
Vitamin D3	25 ng/ml
PT	12
PTT	25
INR	1

WBC: White Blood Cell; RBC: Red Blood Cell; Hg: Hemoglobin; Hct: Hematocrit; Plt: Platelet; Neut: Neutrophils; Lymph: lymphocytes; Mono: Monocytes; cANCA; cytoplasmic Antineutrophil Cytoplasmic Antibodies; pANCA: perinuclear Antineutrophil Cytoplasmic Antibodies; PT: Prothrombin Time; PTT: Partial Thromboplastin Time; INR: International Normalized Ratio

**Fig. 2:**
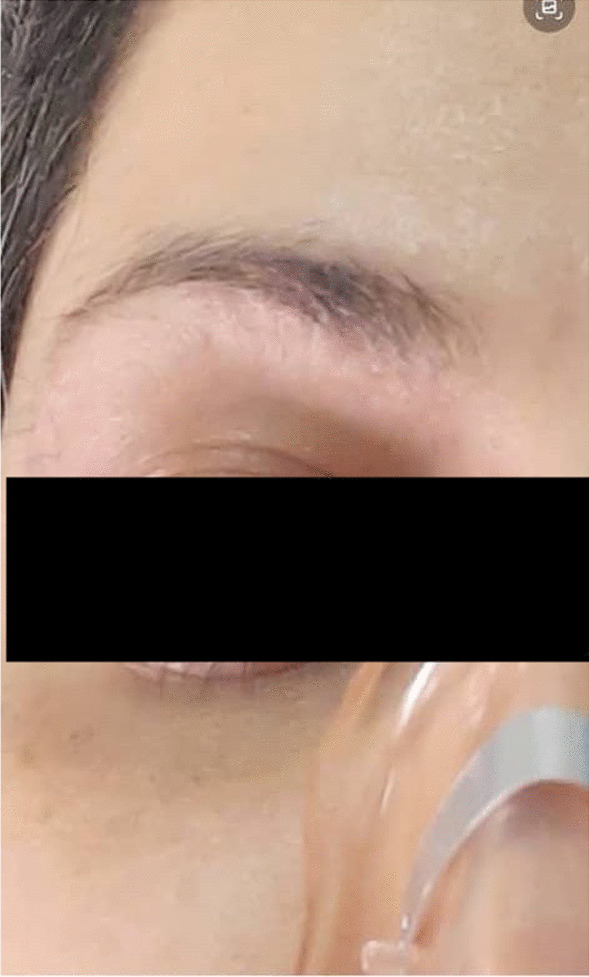
The same patient after treatment

SARS-CoV2 PCR was negative. After excluding the other aforementioned probable causes of chronic dry cough, cough-variant asthma was considered the most likely diagnosis, so an empirical asthma trial therapy was initiated for the patient. The results were surprising. After a 2-week period of treatment, significant improvement of cough was associated with the resolution of the heliotrope-like lesion over the eyes (Fig. 2). The patient has been constantly under close follow-up since the initiation of the therapy without showing recurrence of the signs and symptoms during the treatment.

## Discussion

Asthma is a clinical diagnosis because of its heterogeneous phenotypes without a single diagnostic test to confirm or exclude it [1]. Cough-variant asthma describes an asthmatic phenotype with cough as its sole presentation. The first-line strategy for diagnosis of asthma is spirometry evaluation with 12% and 200 ml improvement in FEV1 from baseline after bronchodilator challenge [8]. However, there is no consensus regarding the challenge of methacholine bronchial provocation test. While some centers perform this test as a main part of the work-up, others do not validate it as a major diagnostic test to assess the condition of the patient [1]. Since airway obstruction in asthma has a variable nature, objective findings in confirming the diagnosis may not be found easily [9]. Even if spirometry and bronchial provocation tests are available, a normal result does not exclude asthma. Therefore, there is no gold-standard test for asthma and its phenotypes [10], and all tests need to be interpreted individually in each patient. The imaging findings of toxic inhalation in HRCT includes bronchial wall thickening, areas of consolidation, bronchiectasis, and the tree-in-bud pattern.

Considering all these facts, our patient revealed increments from baseline in FEV1 after bronchodilator therapy, although the pre-bronchodilator baseline parameters were totally normal. However, methacholine challenge test was negative. The only findings on HRCT imaging were hyperinflation and tree-in-bud opacities, both of which denote toxic inhalation. In addition, the patient’s response to treatment was dramatic. After admission and initiation of optimal asthma treatment, the eye discoloration disappeared as a result of the effective remedy (Figs.  1, 2). According to literature and current guidelines, treating asthma prior to testing reversibility to spirometry is only recommended in clinical emergencies. Our patient showed reversibility in her pulmonary function test even though normal baseline parameters were noted, and her clinical picture was considered urgent. We first took the patient’s detailed history regarding any potential exposure to harmful airborne substances at the time of admission, and she did not mention any exposure except antiseptic solution overuse, so we considered antiseptic solution to be the triggering agent of the patient’s airway hyperreactivity. The fact that she strictly applied social distancing and avoided outdoor as well as crowded areas was indicative of an indoor allergen exposure. Accordingly, we considered the antiseptic solutions the inciting agent and advocated strict avoidance of antiseptic solutions along with medical asthma therapy, resulting in successful control of the disease and alleviation of the signs and symptoms.

This led to the initiation of the treatment. Disappearance of the cough and the eye discoloration were indicative of the correct diagnosis and proper treatment.

## Conclusion

Asthma misdiagnosis is common due to the wide differential diagnosis for cough and also the varying phenotypes of asthma. Since asthma is a clinical diagnosis, a perfect history, physical examination, objective pulmonary tests, and possibly treatment trials are all essential for identifying patients.

## Data Availability

All data and the patient’s consent are available from Dr. Mahsa Rekabi, the first author.

## Abbreviations

COVID-19: Coronavirus disease 2019
HRCT: High-resolution computed tomography
FEV1: Forced expiratory volume in the first second
FVC: Forced vital capacity
Pred: Predicted
PEF: Peak expiratory flow
FEF: Forced expiratory flow
CBC: Complete blood count
cANCA: Cytoplasmic anti neutrophilic cytoplasmic autoantibody
pANCA: Perinuclear anti neutrophilic cytoplasmic autoantibody
PT: Prothrombin time
PTT: Partial thromboplastin time
INR: International normalized ratio
WBC: White blood cell
RBC: Red blood cell
Hg: Hemoglobin
Hct: Hematocrit
Plt: Platelet
Neut: Neutrophil
Lymph: Lymphocyte
Mono: Monocyte
SARS-CoV2: Severe acute respiratory syndrome coronavirus 2
PCR: Polymerase chain reaction
